# Purulent meningitis in X-linked agammaglobulinemia: one case report

**DOI:** 10.3389/fimmu.2026.1704590

**Published:** 2026-03-31

**Authors:** Anyi Ba, Lijiang Wang, Ruifang Ren, Ting C. Zhao, Wenyu Di, Jinggui Song

**Affiliations:** 1 Department of Neurology, The Second Affiliated Hospital of Henan Medical University, Xinxiang, Henan, China; 2 Department of Respiratory and Critical Care Medicine, The First Affiliated Hospital of Henan Medical University, Xinxiang, Henan, China; 3 Department of Neurology, The First Affiliated Hospital of Henan Medical University, Xinxiang, Henan, China; 4 Department of Plastic Surgery, Rhode Island Hospital, Providence, RI, United States; 5 Department of Pathology, The First Affiliated Hospital of Henan Medical University, Xinxiang, Henan, China

**Keywords:** agammaglobulinemia, diagnosis, immunodeficiency, meningitis, next-generation sequencing

## Abstract

X-linked agammaglobulinemia (XLA) is characterized by a triad of primary immunodeficiency, profound hypogammaglobulinemia due to absent antibody production, and a consequent predisposition to severe and recurrent bacterial infections. We employed next-generation sequencing (NGS) on patients with immunodeficiency and their immediate relatives to facilitate the diagnosis and genetic counseling of XLA, thereby improving our understanding of this rare disorder.

## Case presentation

We report one case of an 18-year-old male admitted with a five-day history of fever and headache and diagnosed with purulent meningitis and immunodeficiency. With antimicrobial and dehydration therapy, his symptoms were resolved. Notably, the patient showed a BTK (c.1574G>A, p.R525Q) mutation in chrX:100609675.

## Introduction

X-linked agammaglobulinemia (XLA) is a primary immunodeficiency characterized by profound hypogammaglobulinemia, impaired antibody production, and increased susceptibility to infections (1). Clinical manifestations typically appear in male infants between 3 and 18 months of age. XLA results from mutations in the *Bruton’s tyrosine kinase* (BTK) gene, which encodes a critical signal transduction molecule required for B-cell development. Approximately half of affected individuals have a positive family history, while the remainder arise from *de novo* mutations. Defective BTK function leads to arrested B-cell maturation, resulting in markedly reduced B-cell counts in both blood and tissues, impaired plasma cell differentiation, and consequently, decreased immunoglobulin levels with diminished specific antibody responses. The absence of immunoglobulins essential for opsonization and neutralization predisposes patients to recurrent infections with encapsulated bacteria like *Streptococcus pneumoniae*. *Streptococcus pneumoniae* is a major cause of bacterial meningitis across all age groups worldwide, accounting for approximately 25–50% of reported cases. While mortality rates have declined in developed countries, they remain unacceptably high in developing regions despite the widespread implementation of pneumococcal vaccination programs. Furthermore, survivors frequently experience serious neurological sequelae, including hearing loss, seizures, and cognitive impairment, even after receiving appropriate therapy (2, 3).

## Case presentation

**Figure 1:**
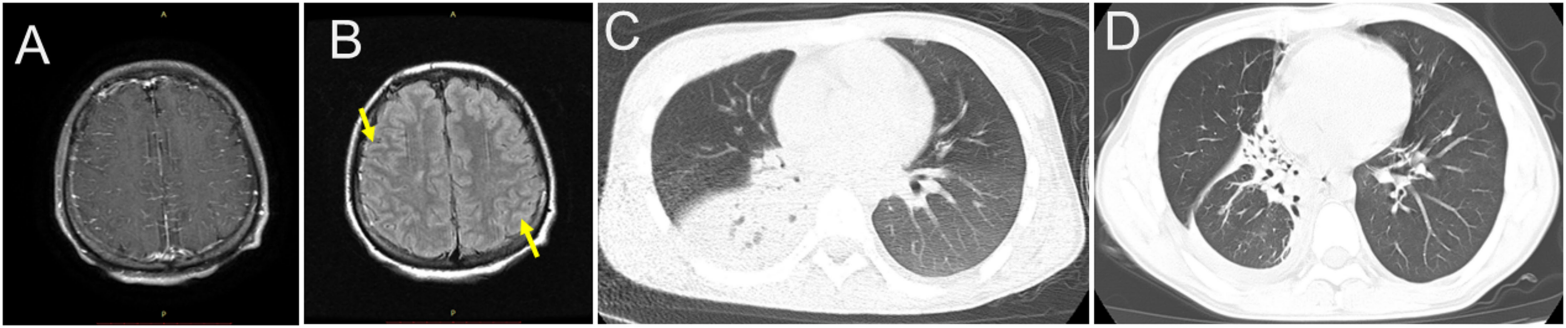
**(A)** Axial contrast-enhanced T1-weighted fat-suppressed MRI demonstrates thin, linear, homogeneous leptomeningeal enhancement along the sulci of the bilateral frontal, temporal, and parietal lobes, consistent with acute meningeal inflammation. No patchy meningeal thickening or abnormal dural enhancement is observed. **(B)** Axial post-contrast T2-FLAIR MRI shows multiple linear hyperintense signals within the cerebral sulci (arrows), suggesting inflammatory exudates in the subarachnoid space. No abnormal enhancement is noted in the cerebellar hemispheres or tentorium. **(C)** Axial chest CT showed consolidation and atelectasis in right middle and lower lobe. **(D)** Axial chest CT showed overall atelectasis in right middle lobe and partial atelectasis in right lower lobe.

**Figure 2:**
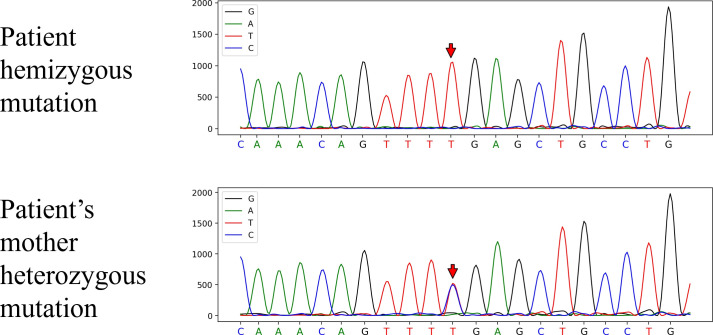
Pedigree demonstrating X-linked inheritance. Whole- exome sequencing identified a hemizygous BTK mutation (c.1574G>A, p.R525Q) located at chrX:100609675.

An 18-year-old male presented with a 5-day history of fever and headache following an upper respiratory tract infection. Five days before the current presentation, the patient developed fever after a chill. The next day, he was evaluated in a local county hospital. Cranial computed tomography (CT) was unremarkable at local hospital. Laboratory test results are shown in **Table 1**. There was with 96.36% neutrophils and an absolute neutrophil count of 35.10 × 10^9/L for blood count. Procalcitonin and interleukin-6 were elevated (**Table 1**). Treatment with intravenous fluids and anti-biotics therapies were administered (specific agents unknown). The fever and headache were unresolved, and the patient was transferred to our hospital. The patient had no pneumococcal or influenza vaccinations prior to hospital admission. The body temperature ranged from 39.0–40.0 °C. The headache was described as a distending pain in the forehead, accompanied by nausea and non-projectile vomiting of gastric contents. There was no history of palpitations, chest tightness, limb convulsions, or altered consciousness. The patient was in good general health but had a history of pneumonia at ages 3 and 6. He was subsequently transferred to our hospital and admitted with a provisional diagnosis of “fever of unknown origin.” He studied in high school and had a younger sister. There was no recurrent infection history for his father, mother and his younger sister. On examination, the temporal temperature was 39.1 °C, the blood pressure 127/76 mmHg. The patient was alert but appeared fatigued. Appetite, sleep, bowel, and urinary functions were normal, with no significant weight loss. Neurological examination revealed normal muscle strength in all four extremities. Mild hypertonia was noted in the upper limbs. The patient was uncooperative with bilateral coordination testing and the Romberg test. Meningeal signs were present, including nuchal rigidity and a positive Kernig’s sign. Laboratory testing revealed leukocytosis and elevated levels of C-reactive protein (CRP), procalcitonin (PCT), and interleukin-6(IL-6); findings of laboratory tests are shown in **Table 1**. Chest CT image showed consolidation and atelectasis in the right middle and lower lobes of the lung (**Figure 1**). Brain magnetic resonance imaging (MRI) showed multiple linear T2-FLAIR hyperintensities are present in the bilateral frontal, temporal, occipital, and parietal sulci, with corresponding linear enhancement on post-contrast imaging. Patchy lesions with iso-intense T1 and mildly hyperintense T2 signals are noted adjacent to the body of the right lateral ventricle and within the centrum semiovale (**Figure 1**). The patient presented with fever, headache, and neck stiffness. MRI revealed linear hyperintense signals along the meninges, suggestive of meningitis. Given the pulmonary and central nervous system involvement and the patient’s critical condition, urgent antimicrobial therapy was initiated with intravenous vancomycin and meropenem, combined with intrathecal vancomycin. Cerebral edema was managed with mannitol, hypertonic saline, corticosteroid, and glycerol fructose. Levetiracetam was administered for seizure prophylaxis, accompanied by symptomatic support including antipyretics and analgesics for headaches. A lumbar puncture was performed, and cerebrospinal fluid (CSF) opening pressure was measured at 200 mmH_2_O. The CSF appeared faintly yellow and slightly turbid. CSF chemistry revealed profoundly elevated total protein and albumin, along with hypoglycorrhachia. The adenosine deaminase (ADA) level was within the normal range (**Table 1**). An India ink stain was negative for *cryptococci*. Further laboratory tests at that time revealed profound reduced immunoglobulin levels: IgG 3.1 g/L and IgM 0.16 g/L (**Table 2**), and IVIG therapy (400 mg/kg for 5 days) was given. Lymphocyte subset analysis demonstrated a significantly low CD19+ B-cell count (4.27 cells/μl) and a CD56+ NK-cell count of 27.92 cells/μl (**Table 2**). On hospital day 4 the temporal temperature decreased to 36.7 °C. On hospital day 8, repeat lumbar puncture was conducted and the CSF opening pressure decreased to 110 mmH_2_O, with colorless and clear appearance. Seral *Mycobacterium tuberculosis* IgG antibody was negative. Metagenomic next-generation sequencing (mNGS, Illumina NextSeq 550) of CSF identified Gram-positive bacteria *Streptococcus pneumoniae* with 30,365 sequence reads and a relative abundance of 76.66%. No pathogenic sequences were detected for fungi, viruses, parasites, mycobacteria, mycoplasma, or chlamydia. This was later confirmed by a blood anaerobic culture, which also grew *Streptococcus pneumoniae*, and a repeat CSF culture further confirmed the presence of the organism. Whole Exome Sequencing (WES, Illumnia, NovaSeq 6000, target region coverage 99.9%, mean depth 118.30, percentage covered at >20X 99.3%) revealed a pathogenic variant in the BTK gene (c.1574G>A), which is absent from ExAC/ESP6500/1000G databases, establishing the diagnosis of XLA. Segregation analysis confirmed maternal inheritance of the mutation (**Figure 2**). On hospital day12, the patient’s symptoms were resolved and then discharged from hospital. The patient was suggested to go to hospital for regular follow-up visits and scheduled IVIG (400mg/kg very 4 weeks) replacement therapy and trough IgG levels should be maintained at least at 5-6g/L. The patient received monthly IVIG infusions but did not adhere to recommended trough IgG monitoring. Twenty-one months later, he was hospitalized locally for a lower respiratory tract infection; chest CT at that time showed improvement compared to findings during his admission to our hospital (**Figure 1**).

**Table 1:** Laboratory data.

Variables	5 days before, local hospital	Day 1, this hospital	Day 4, this hospital	Day 8, this hospital
White-cell count (10^9/L)	36.47	20.41	8.46	–
Neutrophils(10^9/L)	35.10	19.47	5.92	–
Neutrophils (%)	96.36	95.4	70	–
Lymphocytes (10^9/L)	–	0.42	1.88	–
Monocytes (10^9/L)	–	0.51	0.31	–
Eosinophils (10^9/L)	–	0.00	0.33	–
Basophils (10^9/L)	–	0.01	0.02	–
Platelet count (10^12/L)	–	219	335	–
Hematocrit	–	0.386	0.363	–
Hemoglobin (g/L)	–	134	122	–
PCT (ng/m L)	18.831	5.032	0.42	–
IL-6(pg/m L)	127.586	333.74	4.87	–
Total protein (g/L)	–	56.1	57.8	–
Albumin (g/L)	–	30.8	31.1	–
Globulin (g/L)		25.3	26.7	–
ALT (U/L)	–	30	43	–
AST (U/L)	–	18	30	–
C-reactive protein (mg/L)	–	186.65	65.63	–
Lactose(mmol/L)	–	3.23	4.64	–
CSF	–			
Color	–	Faintly yellow	Colorless	Colorless
Appearance	–	Slightly turbid	Slightly turbid	clear
Coagulation	–	Absent	Absent	Absent
Pandy’s test	–	Positive (2+)	Positive (1+)	negative
Nucleated cell count (10^6^/L)	–	1608	970	60
Polymorphonuclear cells (%)	–	79	84	7
Mononuclear cells (%)	–	21	16	93
Chloride^-^(mmol/L)	–	120	119	120.9
Glucose(mmol/L)	–	0.39	1.72	2.00
ADA(U/L)	–	7.1	3.9	1.7
Total protein(mg/L)	–	1069.18	802.22	701.84
ALB(g/L)	–	1019.4	–	–

**Table 2:** Laboratory findings.

Immunoglobulin profile	
IgG	3.1g/L (7.3-15.5)
IgA	0.96 g/L (0.7-3.21)
IgM	0.16g/L (0.51-2.61)
C3	1.33g/L (0.82-1.8)
C4	0.4g/L (0.1-0.4)
Blood flowcytometry analysis	
Total lymphocyte	1232.82 per/uL (1230-3100)
CD8+	490.92 per/uL (360-1250)
CD4+	692.18 per/uL(680-1440)
CD19+B	4.27 per/uL (90-580)
CD56+NK	27.92 per/uL (≧150)

## Discussion

Clinically, XLA is characterized by a near absence of circulating mature B cells, severely decreased serum immunoglobulins, and a consequent predisposition to recurrent and severe infections. Clinical features usually emerge in male infants between 3 and 18 months of age. The condition is caused by mutations in the *Bruton’s tyrosine kinase* (BTK) gene, which encodes a key signaling molecule essential for B-cell development. XLA is caused by mutations in the *BTK* gene, also known as the *Bruton gene*, named in honor of the clinician who first described the disease. Roughly half of patients have a family history of XLA, whereas the remainder result from *de novo* mutations (1). The pathogenic variants affecting the kinase domain, including substitutions at residue R525, are predicted to disrupt B cell receptor (BCR) signaling and block in B cell development at the pre-B cell stage, thereby resulting in absent peripheral B cells, profound hypogammaglobulinemia, and recurrent bacterial infections starting in early childhood (4). Central nervous system (CNS) infections are also frequently observed, affecting 4% to 38% of patients across different centers worldwide and the complications are often fatal (5, 6). Patients with XLA experience recurrent infections and manifestations of immune dysregulation, including autoimmunity, lymphoproliferation, granuloma formation, chronic lung disease, and an increased susceptibility to malignancies (7). Here we report a patient with XLA who developed an meningitis due to *Streptococcus pneumoniae*. Genetic testing identified a hemizygous c.1574G>A mutation in the *BTK* gene. In this patient, the p.R525Q variant identified in our patient likely represents a functionally deleterious mutation consistent with profound humoral immune failure.

In young adults presenting with invasive bacterial meningitis, several alternative immunologic and structural etiologies must be taken into consideration. Complement deficiencies, particularly involving C2, C3, or the terminal components C5–C9, are known risk factors for recurrent meningococcal and pneumococcal infections (8). Structural meningeal defects, such as congenital skull base abnormalities or cerebrospinal fluid leaks, may predispose to recurrent meningitis by providing a direct portal of entry for pathogenic organisms (9). Secondary immunodeficiencies, including those arising from malnutrition, immunosuppressive therapy, or systemic illness, must also be excluded. Functional or anatomic asplenia increases susceptibility to encapsulated organisms, most notably *Streptococcus pneumoniae* (10). In addition, other primary humoral immunodeficiencies, such as common variable immunodeficiency (CVID) and selective antibody deficiency, can manifest with recurrent sinopulmonary and invasive bacterial infections (11). The simultaneous identification of invasive *Streptococcus pneumoniae* infection in both the pulmonary system and the central nervous system strongly suggests hematogenous dissemination, a mechanism facilitated by profound humoral immune deficiency in XLA. In this patient, the markedly reduced serum immunoglobulin levels, near absence of circulating B cells, history of early-onset recurrent infections, and the presence of a confirmed pathogenic *BTK* variant collectively provide compelling evidence for XLA, making it the most comprehensive and unifying diagnosis in this patient.

Early diagnosis and prompt treatment are critical in preventing infections and secondary complications, highlighting the importance of newborn screening (NBS) for XLA. However, identifying these X-chromosomal pathogenic variants enables carrier detection, which is challenging in the absence of a known family history. Patients identified early typically experience fewer severe or recurrent infections and require fewer hospitalizations (12). Although XLA predisposes individuals to a broad spectrum of infections, enteroviral disease remains among the most severe and clinically devastating complications. In patients with XLA, enteroviral infections most commonly present as dermatomyositis or chronic enteroviral meningoencephalitis (CEMA), the latter representing a progressive and potentially fatal central nervous system complication. However, the diagnosis of CEMA is often challenging. Diagnostic difficulties arise from the limited sensitivity of CSF assays for enterovirus detection and delays in recognizing early neurologic symptoms. Maintaining adequate trough levels of intravenous immunoglobulin (IVIG) replacement therapy can effectively reduce bacterial infections, but such treatment does not appear to confer sufficient protection against enteric viral infections (13). Accordingly, patients with XLA who develop progressive neurologic deterioration should be strongly evaluated for CEMA, and a high index of suspicion should be maintained until alternative etiologies have been carefully excluded (14, 15). The patient in our case did not test the specific IgG including rubella and tetanus due to regular vaccination during childhood. Beyond neurologic complications, patients with XLA commonly experience recurrent sino-pulmonary infections as well as gastrointestinal infections; however, the frequency and severity of these manifestations vary among individuals. These infections are most often caused by encapsulated pyogenic bacteria, particularly *Streptococcus pneumoniae*, *Haemophilus influenzae* type B, *Streptococcus pyogenes*, and *Pseudomonas* species. In our case, the clinical presentation was consistent with acute bacterial meningitis, characterized by fever, headache, increased muscle tone in both upper limbs, neck stiffness, and a positive Kernig’s sign, rather than features typical of chronic viral encephalitis, such as progressive motor and cognitive impairment or neurodegenerative manifestations. Moreover, the same strain of *Streptococcus pneumoniae* was simultaneously identified in both blood and cerebrospinal fluid samples. In addition to infectious complications, patients with XLA may also develop inflammatory manifestations such as arthritis, inflammatory bowel disease, or other immune-mediated disorders, which occur more frequently than in the general population (16). In one study, sepsis was identified as the most common clinical presentation (17). The children affected carried the same BTK variants that had been identified in the umbilical cord samples of their maternal uncles. Examination of preserved umbilical cords may therefore serve as a useful tool in establishing a family history of inborn errors of immunity (IEIs) (18).

The cornerstone of management is immunoglobulin replacement therapy. In addition, general protective measures, including infection avoidance and immunization with inactivated vaccines, are recommended. Immunoglobulin therapy reduces the frequency of infections and hospitalizations, helps prevent long-term pulmonary complications, and lowers the risk of systemic enteroviral infection (7). Over the past two decades, the prognosis of XLA has improved substantially, with many patients now surviving into adulthood due to earlier diagnosis, timely initiation of immunoglobulin replacement therapy, and advances in infection management. Previous studies have reported that the annual mortality rate has decreased from 17–25% to approximately 1%. Nevertheless, chronic lung disease and sepsis remain the leading causes of death, and more than half of patients succumb to pulmonary complications before the age of 45.

Although immunoglobulin replacement therapy has significantly improved both life expectancy and quality of life, it is neither curative nor without limitations. The high cost of IVIG in many countries, the lifelong need for regular treatment and follow-up, and its inability to fully protect against certain pathogens due to the absence of IgM and IgA represent important challenges.

Alternative therapeutic strategies include hematopoietic stem cell transplantation (HSCT) and gene therapy using viral vectors. While these approaches remain under investigation and require further refinement, gene therapy holds particular promise as a potential curative option, with the capacity to permanently correct the defective *BTK* gene (19, 20).

## Conclusion

This case highlights that central nervous system infections caused by *Streptococcus pneumoniae* are a rare yet treatable complication in individuals with XLA. It further stresses the importance of early recognition of XLA to facilitate timely therapeutic intervention.

## Data Availability

The original contributions presented in the study are included in the article/supplementary material. Further inquiries can be directed to the corresponding authors.
